# Bone invading NSCLC cells produce IL-7: mice model and human histologic data

**DOI:** 10.1186/1471-2407-10-12

**Published:** 2010-01-12

**Authors:** Ilaria Roato, Davide Caldo, Laura Godio, Lucia D'Amico, Paolo Giannoni, Emanuela Morello, Rodolfo Quarto, Luigi Molfetta, Paolo Buracco, Antonio Mussa, Riccardo Ferracini

**Affiliations:** 1 CeRMS (Center for Experimental Research and Medical Studies) University of Turin and A.O.U. San Giovanni Battista, Turin, Italy; 2 Department of Orthopaedics, A.O.U. San Giovanni Battista, Turin, Italy; 3 Department of Experimental Medicine (DIMES), University of Genoa, Italy; 4 Department of Pathology, A.O.U. San Giovanni Battista, Turin, Italy; 5 ABC Advanced Biotechnology Center, Genoa, Italy; 6 Department of Animal Pathology, University of Turin, Grugliasco, Italy; 7 University of Genoa - Department of Motor Sciences, Italy; 8 Department of Medical Oncology, A.O.U. San Giovanni Battista, Turin, Italy

## Abstract

**Background:**

Bone metastases are a common and dismal consequence of lung cancer that is a leading cause of death. The role of IL-7 in promoting bone metastases has been previously investigated in NSCLC, but many aspects remain to be disclosed. To further study IL-7 function in bone metastasis, we developed a human-in-mice model of bone aggression by NSCLC and analyzed human bone metastasis biopsies.

**Methods:**

We used NOD/SCID mice implanted with human bone. After bone engraftment, two groups of mice were injected subcutaneously with A549, a human NSCLC cell line, either close or at the contralateral flank to the human bone implant, while a third control group did not receive cancer cells. Tumor and bone vitality and IL-7 expression were assessed in implanted bone, affected or not by A549. Serum IL-7 levels were evaluated by ELISA. IL-7 immunohistochemistry was performed on 10 human bone NSCLC metastasis biopsies for comparison.

**Results:**

At 12 weeks after bone implant, we observed osteogenic activity and neovascularization, confirming bone vitality. Tumor aggressive cells implanted close to human bone invaded the bone tissue. The bone-aggressive cancer cells were positive for IL-7 staining both in the mice model and in human biopsies. Higher IL-7 serum levels were found in mice injected with A549 cells close to the bone implant compared to mice injected with A549 cells in the flank opposite to the bone implant.

**Conclusions:**

We demonstrated that bone-invading cells express and produce IL-7, which is known to promote osteoclast activation and osteolytic lesions. Tumor-bone interaction increases IL-7 production, with an increase in IL-7 serum levels. The presented mice model of bone invasion by contiguous tumor is suitable to study bone-tumor cell interaction. IL-7 plays a role in the first steps of metastatic process.

## Background

Lung cancer is a major cause of cancer-related deaths [1]. More than 90% of deaths due to lung cancer can be attributed to metastases [2], which are frequently observed at the time of diagnosis. After liver and brain, bone represents the most common target organ [3]. Osteolytic bone metastases worsen prognosis, causing morbidity [4] and also having substantial financial implications for the health-care providers [5]. The low success rate in treating lung cancer depends on a still incomplete understanding of the metastatic process and on the lack of sensitive markers to predict and monitor tumor progression [6].

The role of IL-7 in diseases characterized by bone loss[7] and in bone metastases [8-10] has been deeply investigated by us and others. Particularly, we identified IL-7 as a potential marker of Non-Small Cell Lung Cancer (NSCLC) progression to bone. The role of IL-7 production by tumor cell is still elusive, but it may be pivotal in metastasis pathogenesis. In physiological conditions IL-7 is usually subject to strict regulation, while production is rarely affected. Disfunction of this fine regulation can only be found in severely impaired osteoimmunologic situations [11-13].

In the last years, important advances provided new animal models of different human cancer metastasis to human tissues [14-16]. Some models gave important contributions to the disclosure of the osteotropic mechanisms. They involved the implant of both human tumor cells and human bone as a metastatic target in non-obese diabetic severe combined immunodeficiency (NOD/SCID) mice, allowing the study of the cell-cell interactions within all human tissues at the target site. In this work, a human-in-mice model was adopted; a human bone implant was susceptible to metastatic spread, as previously described by Kuperwasser et al. to study spread through circulation from orthotopic site [14]. We rather focused on bone-tumor interaction, studying direct invasion by a tumor mass growing close to the engrafted bone. The tumors derived from subcutaneous injection of a human NSCLC cell line, A549. We studied the IL-7 production by bone invading cells and we tested the IL-7 expression in bioptic tissues from human secondary bone lesions, due to NSCLC.

## Methods

### Cell culture

A NSCLC cell line with osteotropic characteristics, the A549, was purchased from the American Type Culture Center (ATCC). Cells were cultured in F-12K Medium, Modified 2 mM L-glutamine e 1500 mg/L sodium bicarbonate (ATCC), 10% fetal bovine serum (FBS) (Sigma-Aldrich, St Louis, MO) with antibiotics, according to manifacturer's conditions. Sub-confluent cells were washed by PBS and harvested by trypsinization.

### Animals and surgery

A colony of 18 NOD/SCID 7-week-old female mice (Charles River Laboratories, Calco, Milan) were housed under aseptic sterile conditions. Experimental animals were treated in compliance with the actual national and international guidelines (the Italian legislative decree 116/92 and the European Community Directive 86/609 CEE) and in accordance to the authorization provided by Italian Ministry of Health (as of D.M. 44/1994-A and subsequent integrations).

Mice were given autoclaved food and water ad libitum. A 0.5 cm^3 ^cube-shaped human bone implant, obtained from the discarded head bone of an adult patient submitted to total joint replacement (after the patient's informed consent), was immediately transplanted sub-cutaneously in the left flank in all animals. Surgery was performed in sterile conditions and in general anaesthesia. Animals received antibiotics (Enrofloxacin 2.5% 1 ml/1 L) in the drinking water up to 2 weeks following all surgical procedures.

The bone implants were allowed to engraft in the mice for 4 weeks; then mice were divided in three groups of six animals each, as follows: a) mice subjected only to bone implant (control); b) mice injected with A549 cells close to the bone implant (group #1); c) mice injected with A549 cells in the opposite flank to the bone implant (group #2). In particular, 1 × 10^6^of cells were resuspended in PBS and Matrigel 1:3 (BD Biosciences, Bedford, MA) and injected in volume of 40 μL sub-cutaneously, using a 25-gauge needle.

Eight weeks after cancer cell injection, mice were euthanized; primary tumors, bone implants, lungs and spleens were excised and processed for immunohistochemistry and other analyses.

### Histology and immunohistochemistry

Immunohistochemistry was performed on tissues fixed in 10% neutral buffered formalin and bone tissues were decalcified with EDTA treatment until soft. Tissues were embedded in paraffin and 4 micron sections were deparaffinized, rehydrated through graded alcohols, and subjected to antigen retrieval for immunohistochemistry. Sections were stained for H&E to study the morphology and incubated with mouse monoclonal antibodies against human-specific CD34, clone QBEnd/10 (NeoMarkers, Fremont, CA) and human IL-7 (R&D Systems, Abingdon, UK). IL-7 staining was performed also on 10 human biopsies derived from bone metastasis by NSCLC. Research on human specimen was carried out in compliance with the Helsinki Declaration. IL-7 expression in mice and human tissues was quantifed based on IL-7 staining intensity.

### Fluorescence-activated cell sorting analysis and ELISA

FACS analysis was performed on whole NOD/SCID mice spleens. Spleen tissue was dissociated with forceps and cells were re-suspended in D-MEM with 10% FBS. Cells were passed through a 40 μm cell strainer and re-suspended in red blood cell lysis buffer. Two million cells were incubated with antibodies specific for human IgG-phycoerythrin (PE). Spleen cells were analyzed on a FACSCalibur system to identify the human IgG-PE positive cell population after gating out PI positive cells. Samples were analyzed in a FACsCalibur instrument and elaborated by CellQuest software.

To dose serum IL-7 levels we performed an ELISA assay, according to the manifacturer's instructions (R&D Systems, Abingdon, UK). The kit sensitivity was 0.27-8.7 pg/mL.

## Results

### Analysis of viability and activity of implanted human bone

**Figure 1:**
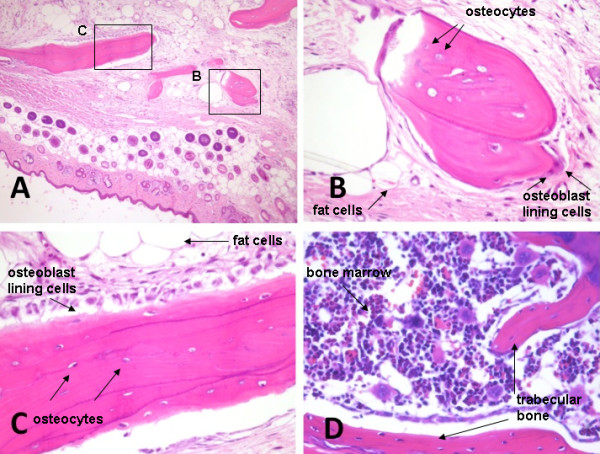
**Histologic analysis of human bone implants**. Representative H&E-stained section of human bone fragment implanted subcutaneously in NOD/SCID mice 12 weeks post implantation (A). Magnifications show newly synthesized bone, osteocytes, fat cells, areas of solid mineralized bone and osteoblast lining cells, as labelled by the arrows (B and C). Human bone marrow remains localized in the marrow spaces, as indicated in D. Magnification: A 100×, B 250×, C 250×, D100×.

**Figure 2:**
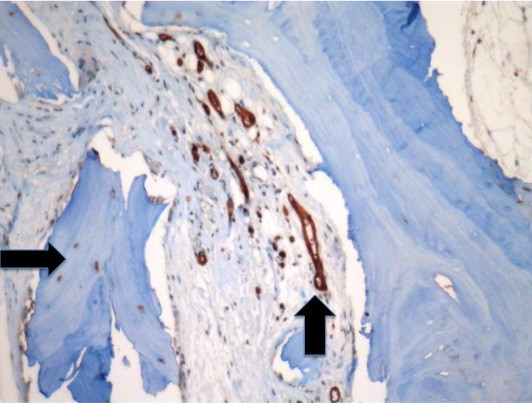
**Immunohistochemical staining of human bone implants**. Immunohistochemistry was performed on serial sections using human-specific CD34 antibodies to delineate human blood vessels, which are shown within and around bone xenografts (arrows). Magnification 250×.

A human bone was implanted in NOD/SCID mice, according to the previously described method [14]. Twelve weeks post-implantation the human bone implants, mouse spleens, lungs and blood were collected from the xenografted hosts. Survivorship and percentage of bone and lung metastatic invasion are listed in table 1. H&E staining of bone implants showed mineralized bone and stromal cells (Fig. 1A). In detail, osteocytes as well as fat cells, areas of solid mineralized bone, osteoblast lining cells throughout these areas, bone forming activity (Fig. 1B-C) and bone marrow (Fig. 1D) were observed *in situ*. The vascularization of the grafts was examined and neo-angiogenesis surrounding the implanted bone was detected. (To determine the species origin of endothelial cells in blood vessels supplying the bone implants, sections were examined by immunohistochemistry using human-specific CD34 antibodies). The presence of human derived blood vessels was confirmed by CD34 staining of human blood vessels (Fig 2), while mouse vessels were not stained. The human vasculature remained viable within bone for periods of 12 weeks.

**Table 1:** mouse groups and human biopsies summary data

	Survivorship	Bone metastatic invasion	Intensity of IL-7 staining	Metastasis in lung tissue
**Control**	5/6 (83%)	0	+ (stromal cells)	0

**Group #1**	5/6 (83%)	4/5	+++ (tumor cells and stromal cells)	micro- metastasis 1/5

**Group #2**	6/6 (100%)	0	+ (stromal cells)	0

**10 Human biopsies**	-	10/10	+++	-

**Control **indicates mice subjected only to bone implant; **group #1 **indicates mice injected with A549 cells close to the bone implant; **group #2 **indicates mice injected with A549 cells in the opposite flank to the bone implant.

To further demonstrate that human bone marrow precursors were functional, we analyzed by FACS the presence of human IgG in the host mouse spleens, showing their presence in mice with bone implant, whereas no IgG were found in the control mouse spleens, 12 weeks after human bone implantation (data not shown).

### NSCLC cells invade the implanted human bone

**Figure 3:**
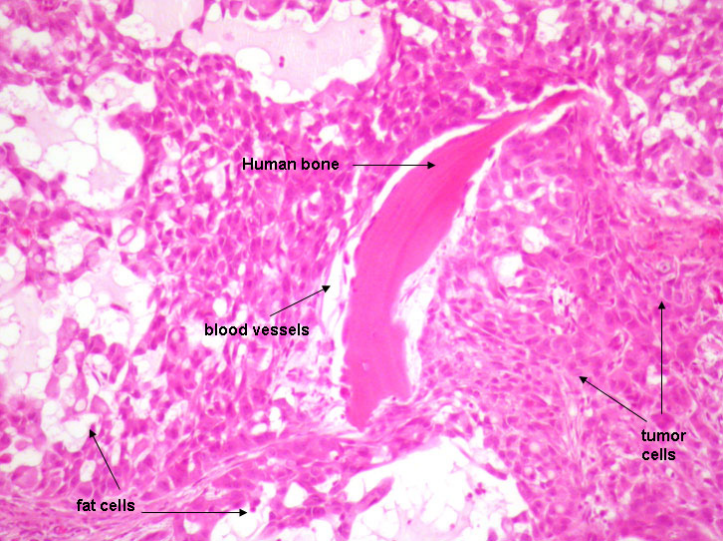
**Histologic analysis of A549 metastases in human implanted bone**. H & E-stained sections show large areas of invading tumor cells surrounding live human bone, with presence of fat cells, blood vessels, and osteoblasts, as labeled by the arrows. Magnification 100×.

The A549 is a NSCLC cell line with osteotropic ability. A rapid tumor growth was obtained at the implantation site in all animals. When tumor cells were injected close to the bone chip, the tumor invaded the normal implanted human bone, disrupting it. Metastases were not observed in the group where tumor cells were injected in the contralateral flank. H&E staining of bone implant with tumors showed large areas of invading tumor cells surrounding live human bone, with presence of fat cells, blood vessels, and osteoblasts (Fig 3).

### NSCLC bone invading cells express IL-7

**Figure 4:**
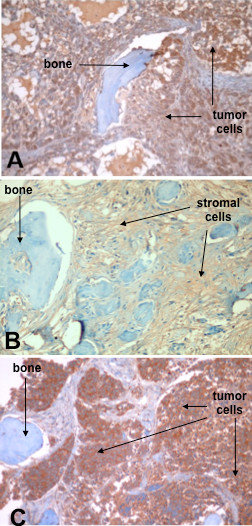
**IL-7 expression by NSCLC bone invading cells in mouse and human biopsies**. A strong IL-7 level expression is shown by tumor cells in invaded bone (A). In contralateral bone, IL-7 level expression is detectable on stromal cells and it is lower than in bone invaded by tumor cells (B). In human biopsies, IL-7 is highly expressed by bone invading cells (C). Magnification 100×.

IL-7 expression by A549 cells was evaluated, showing a high IL-7 expression by tumor cells of epithelial origin. IL-7 expression in tumor masses was observed in both groups. The analysis of bone invaded by tumor cells and bone contralateral to the tumor mass, showed a different intensity of IL-7 expression. In detail, bone invaded by tumor cells showed a stronger level of IL-7 expression than contralateral bone, where IL-7 was expressed only by stromal cells (Fig. 4A, B). In human biopsies, IL-7 was highly expressed by bone invading cells (Fig. 4C). The intensity of IL-7 expression is reported in Table 1.

### Serum IL-7 is high in mice with bone invasion

Since we previously demonstrated that serum IL-7 levels are higher in NSCLC patients with bone metastases than in patients without bone lesions, we tested the IL-7 production in the two groups of mice, by dosing serum IL-7.

**Figure 5:**
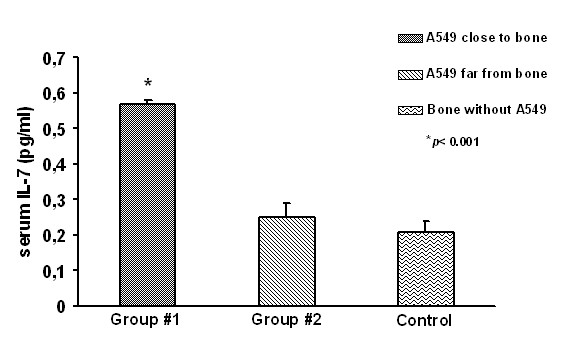
**Serum IL-7 levels in mice**. In mice injected with A549 cells close to the bone implant IL-7 is significantly higher than in mice injected with A549 cells in the opposite flank, *p *< 0.001. There was not significant difference between opposite flank and the control group without tumor cells.

Significantly higher levels of IL-7 were found in mice injected with A549 cells close to the bone implant compared to mice injected with A549 cells in the opposite flank (0.57 ± 0.01 and 0.25 ± 0.03 pg/ml, respectively; *p *< 0.001), while no significant difference was found between opposite flank and control group without tumor cells. The IL-7 serum value in the control group was 0.21 ± 0.03 pg/ml (Fig. 5).

## Discussion

One barrier to the disclosure of cancer osteotropism mechanism is the lack of animal models that reflect the complex biology of the metastatic process in humans [17]. Some models are limited to intra-cardiac or intravenous (tail vein) injection, where cancer cells are directly seeded into circulation, bypassing the early steps of the metastatic process [18-21]. The result is a process that may be quite different from what happens in humans, where tumor cells undergo a phenotype selection before and during metastatic spread to the circulation. Moreover, human tumor cells interact with mouse tissue at the target site possibly introducing a further bias.

Recently, new animal models provided important advances for investigations of human bone metastasis [14-16]. These models involve implant of both human tumor cells and a xenograft of human tissue as metastatic target in NOD/SCID mice, allowing cell-cell interactions within all human tissues at the target site and a selection of the metastatic phenotype.

In the models where metastatic target is human bone tissue, the Kuperwasser method [14] worked only in a selected cell line over thirteen tested. No molecular explanation or hypotesis was provided and the model was never used by other authors. Another model, proposed by Yang and colleagues [16], required the implant of bone tissue at the site of primary tumor seeding in order to obtain metastasis at a target bone site, a very peculiar condition, quite different from conventional human metastatic process.

The model proposed in this study is based on human bone invasion by a contiguous human tumor mass; it doesn't replicate the comprehensive metastatic process (evasion from primary tumor, vessel invasion and survival in the blood circulation), rather focuses on cancer cell bone invasion capability. It allowed us to bypass technical difficulties related to obtaining NSCLC metastases through blood circulation. The opposite flank injection group was useful for serum IL-7 level comparisons, in order to discriminate production related to bone invasion from production by tumor mass alone. To the author's knowledge, this is the first human-in-mice NSCLC bone invasion model described in literature. The general validity of this model is supported by results on bone vitality and implanted tumor growth. The specific validity for studies on tumor-bone interactions in metastasis is highlighted by the histologic and cytokine production analyses. Bone invasion appeared to have the same histological characteristics of human metastasis, such as bone resorption, neo-apposition and tumor nested within the bone tissue. The immunohistochemical staining of bone invaded by NSCLC cells, showed a strong expression of IL-7; the same result was obtained by the staining performed on human tissues from bone biopsies of patients affected by NSCLC bone metastasis. Staining for IL-7 of the bone implanted contralateral to the tumor showed stromal cells positive for IL-7 expression, as expected and according to the literature [22,23]. In the past, the Authors showed that increase in IL-7 serum levels relates to NSCLC bone metastasis but couldn't demonstrate the capability of bone metastatic cells to directly produce IL-7 [10]. Here we show that NSCLC bone invading cells expressed and produced detectable quantities of IL-7 both in human metastasis and in our model. The IL-7 expression by tumor mass (in mice with contralateral bone) and by bone stromal cells did not lead to high serum IL-7 levels. In fact, the highest IL-7 serum levels were detected in the experimental group where tumor cells invaded the human bone target. Thus, interactions between tumor cells and bone increased the IL-7 serum levels, as demonstrated in patients [24].

We speculate that the mechanism underlying interaction between cancer cells and bone closely resembles the metastatic mechanism in humans, therefore this model might be valuable and considered for further testing.

## Conclusions

We demonstrated that bone invading cells directly express and produce IL-7, which is known to promote osteoclast activation and osteolytic lesions. Both tumor cells and bone stroma can produce IL7 but cross-talk between the two increases production, as shown both in humans and in the described mice model. Our mice model of bone invasion by contiguous tumor mass showed similar histology and IL-7 patterns compared to human pathology. We believe it to be suitable for studies on bone-tumor cell interaction, being both realistic and technically feasible compared to other models.

We believe that new studies on the first steps of metastatic process and on the role of IL-7 are due, considering together the fine mechanism through which IL-7 is regulated, its role in pathologies characterized by bone loss and the sudden intense increase in IL-7 production when bone is invaded by tumor cells.

## Competing interests

The authors declare that they have no competing interests.

## Authors' contributions

IR: conception and design of the study, analysis and interpretation of data, carried out part of laboratory assessment and the draft of the manuscript. DC: conception and design of the study, analysis and interpretation of data, carried out part of the experimental surgery and the draft of the manuscript. LG: carried out and analysis of immunohistochemical staining. LD: carried out laboratory assessment. PG: was responsible for animal care and surgical procedures. EM: carried out part of the experimental surgery. QR: critical revisal of the manuscript. LM: interpretation of data. PB: critical revisal of the manuscript. AM: critical revisal of the manuscript. RF: interpretation of data, critical revisal of the manuscript. All authors read and approved the final manuscript.

## Pre-publication history

The pre-publication history for this paper can be accessed here:

http://www.biomedcentral.com/1471-2407/10/12/prepub
